# Insights from new in vivo models of *TREM2* variants

**DOI:** 10.1186/s13024-023-00609-4

**Published:** 2023-04-05

**Authors:** Nimansha Jain, David M. Holtzman

**Affiliations:** 1 Department of Neurology, Washington University School of Medicine, St. Louis, MO USA; 2 Hope Center for Neurological Disorders, Washington University School of Medicine, MO St. Louis, USA; 3 Knight Alzheimer’s Disease Research Center, Washington University School of Medicine, MO St. Louis, USA

**Keywords:** Alzheimer’s disease, TREM2, Amyloid-beta, Microglia

Recent genetic studies have highlighted the central role of the innate immune system in Alzheimer’s disease (AD) by identifying several risk variants in genes that are exclusively expressed within microglia in the brain. Most notably, rare variants, such as the R47H, R62H and H157Y mutations, in the Triggering Receptor Expressed on Myeloid Cells 2 (TREM2) gene increase risk for late-onset AD [1]. TREM2 is a cell surface receptor expressed specifically by microglia in the brain. TREM2 signaling occurs through the immunoreceptor tyrosine-based activation motif (ITAM)-containing adaptor DAP12 and its function supports diverse processes such as phagocytosis and clustering around debris, increased metabolic function, and lipid metabolism [2]. Mounting evidence suggests that TREM2 function is age, context, and disease dependent. With the development of mouse models that better align to human TREM2 mutations and human *Trem2* expression levels, there is more data available on how the human *Trem2* gene functions in the pathophysiology of AD.

In two research articles recently published in *Molecular Neurodegeneration*, Tran et al. and Qiao et al. push the field of TREM2 biology forward through two uniquely and newly generated mouse models. Tran et al. explore the response of a new *Trem2* R47H knock-in mouse model without cryptic splicing to acute demyelination following administration of cuprizone and within the context of early and late amyloidosis [3]. Qiao et al. explore a newly generated *Trem2* H157Y knock-in mouse model to study the role of the H157Y variant within the context of amyloidosis [4]. Both studies can potentially help bridge some current limitations to presently available TREM2 mouse models.

Several approaches have been utilized to create *Trem2*^R47H^ mouse models, including the use of CRISPR/Cas9 and through bacterial artificial chromosome (BAC) technology. It has been reported that *Trem2*^R47H^ knock-in models created through CRISPR exhibit aberrant splicing and reduced expression levels of *Trem2* mRNA [5–7]. Other models using the BAC transgene technology containing either the common variant (CV) or the R47H variant of human TREM2 crossed to 5XFAD mice with mouse TREM2 knocked out show that R47H mice have lower levels of isoform 1 and isoform 2 *Trem2* mRNA expression, but similar levels of human TREM2 protein in myeloid cells, compared to CV mice [8]. Tran et al. show that the newly designed *Trem2*^R47H NSS^ (Normal Splice Site) model compared to the *Trem2*^R47H CSS^ (Cryptic Splice Site) model display expression of *Trem2* at similar levels to wild type (WT) mice without evidence of cryptic splicing products (Fig. 1A).

**Fig. 1:**
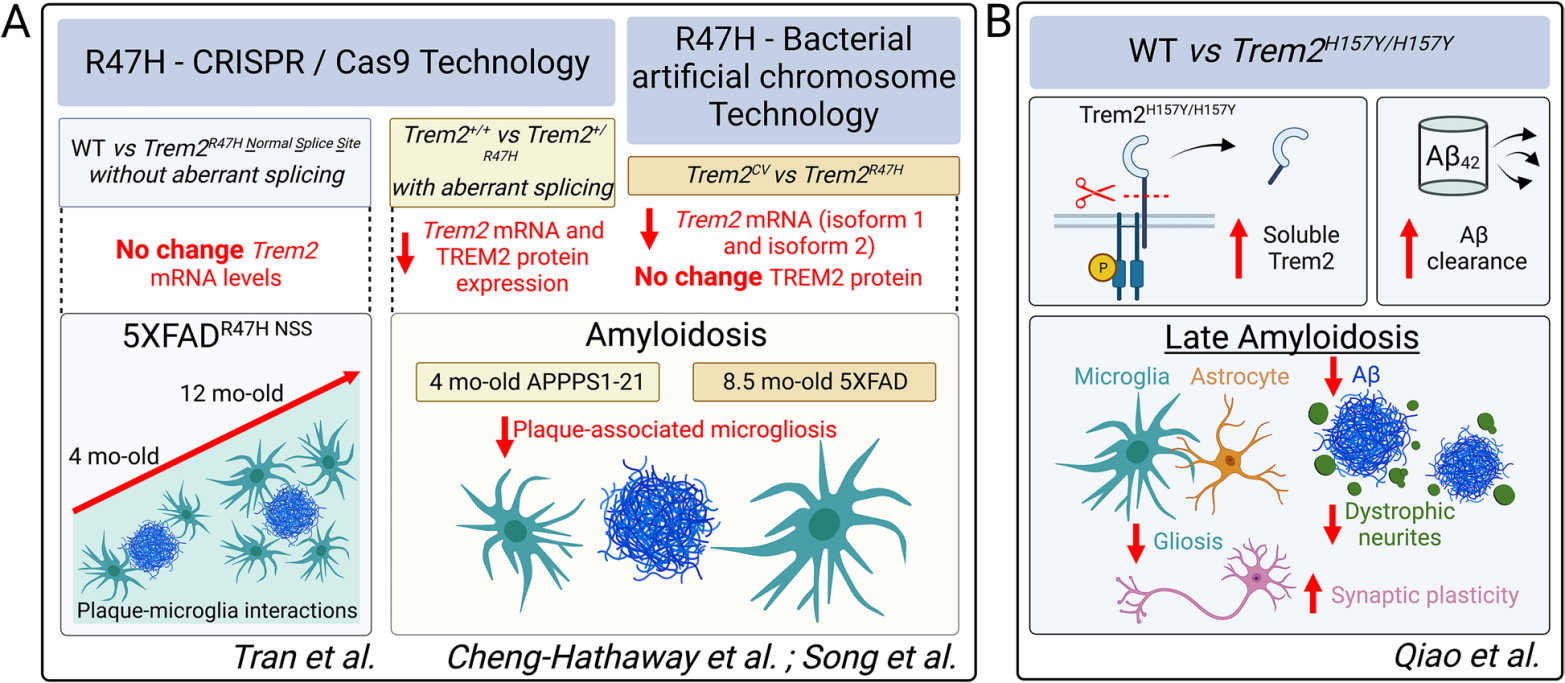
Insights from New in vivo Models of *TREM2* Variants. **A**. *Trem2*^R47H^ mouse models created through CRISPR/Cas9 and through bacterial artificial chromosome technology and their effects on Trem2 mRNA levels and protein levels if assessed. Highlight of plaque and microglia interactions in *Trem2*^R47H^ mouse models. **B**. The H157Y variant in a mouse model of amyloidosis increased levels of soluble Trem2 and increased Aβ clearance. During late amyloidosis (8.5 mo-old), Qiao et al. observed a reduction in amyloid burden, dystrophic neurites, and gliosis. This was paralleled with enhanced synaptic plasticity

Following administration of cuprizone, *Trem2*^R47H NSS^ mice showed similar levels of microglial activation as *Trem2*^R47H CSS^ and wild type mice, but much higher levels compared to *Trem2*^KO^ mice. Although, there is microglial activation in *Trem2*^R47H NSS^ mice, these mice showed increased oligodendrocyte gene expression loss like that seen in the *Trem2*^KO^ mice. In response to cuprizone administration, the study demonstrates that the R47H variant in *Trem2*^R47H NSS^ mice does not appear to function as a loss of function allele in terms of a microglial response but does phenocopy the exacerbated oligodendrocyte damage seen in the KO condition. Tran et al. also did a thorough analysis of amyloid-β (Aβ) plaques in 5XFAD mice and showed that Aβ levels and compaction are brain region-, age- and sex- specific in *Trem2*^R47H NSS^ mice. Largely, they showed that the *Trem2*^R47H NSS^ mice exhibited suppressed microglial activation early in disease that conversely shifted to a unique interferon signature with age. The earlier stage also showed impaired microglia-plaque interactions and less compacted plaques with increased dystrophic neurites. This impairment is lost at 12 months of age (Fig. 1A). The effects seen during early amyloidosis on microglial activation are similar to that seen in a *Trem2*^R47H^ knock-in mouse model that has lower expression of Trem2, where 4-month-old APPPS1-21 expressing the R47H variant showed lower levels of plaque associated myeloid cells [5]. These results vary somewhat from what was observed in the BAC transgene model system studying an age between 4 and 12 months in 8.5-month-old 5XFAD mice, in which the CV but not the R47H variant augmented plaque-associated microgliosis and enhanced microglial activation with minimal changes to Aβ levels between those groups [8]. An interesting finding that warrants further exploration is the effect of the R47H variant in the *Trem2*^R47H NSS^ mice on increased dystrophic neurites, NfL levels, paralleled with a surprising protective effect against plaque-induced long-term potentiation and synaptic deficits.

In vivo understanding of a more rare *Trem2* mutation, H157Y, is limited. To address this, Qiao et al. explore a newly generated *Trem2* H157Y knock-in mouse model within the context of amyloidosis. The homozygous (hom) *Trem2*^H157Y/H157Y^ mice exhibit increased shedding of TREM2 as they displayed higher levels of soluble TREM2 (sTREM2) in serum and brain compared to the heterozygous (het) and WT groups. The hom Trem2H157Y/H157Y mice displayed enhanced Aβ clearance and reduced total Aβ burden at the late stage of Aβ development, but minimal changes in the early stage of Aβ deposition. Interestingly, there were no changes in synaptic markers like synaptophysin but an enhancement in paired-pulse facilitation and strengthened long-term potentiation recordings in the hom *Trem2*^H157Y/H157Y^ mice compared to WT mice. This data supports a beneficial effect of the H157Y mutation on synaptic plasticity, presynaptic function, and on amyloid burden during the later stage of amyloidosis (Fig. 1B). Finally, Qiao et al. demonstrate the late stage hom *Trem2*^H157Y/H157Y^ mice have reduced gliosis and decreased Trem2 signaling compared to WT mice.

Both studies indicate that identifying the optimal time window to intervene in a disease like AD will be key to reach treatment effectiveness when targeting TREM2 function. Additionally, moving forward we may need to consider the specific type of *Trem2* mutation the patient carries as not all have the identical effects on AD pathology. As Qiao et al. demonstrate, the *Trem2* H157Y mutation did not affect Aβ pathology at the early stage of Aβ development but reduced Aβ burden and increased Aβ clearance at the later stage of amyloidosis. The in vivo effects of the H157Y mutation on Aβ burden differ from what has been observed in vivo utilizing amyloidosis models with the R47H mutation [5, 8, 9]. Furthermore, Tran et al. showed that the *Trem2*^R47H NSS^ mice exhibit suppressed microglial reactivity early in amyloidosis that conversely shifts to a unique interferon signature with age and amyloid accumulation. These effects at the early and later times assessed by Tran et al. also differed based on brain region with noticeable sex differences.

These two studies and others also highlight the importance of understanding the type of pathology and pathology severity in the brains of patients with AD when therapeutically targeting TREM2 function. Neuroanatomically, fibrillar Aβ deposition occurs first and most severely in regions such as the precuneus and frontal lobes [10] during the presymptomatic phase of AD. As Aβ pathology progresses in humans, tau pathology spreads from the medial temporal lobe into the limbic cortex and neocortex. Aβ plaques also mediate local tau seeding in dystrophic neurites and contribute to the formation and spread of p-tau in neuritic plaques and neurofibrillary tangles in mice [11]. Further studies will be necessary to elucidate the effects of TREM2 variants and targeting TREM2 function within the context of Aβ-induced tau seeding and spreading as well as in the phase of tauopathy that is closely linked with neurodegeneration. Another consideration onward from a therapeutic perspective will be to characterize the effect of TREM2 agonism and antagonism. We recently showed that in a mouse model of the phase of AD when Aβ is driving tau seeding and spreading, chronic TREM2 activation with a TREM2 antibody in the absence of amyloid removal stimulated a disease associated microglia phenotype but surprisingly increased tau seeding and spreading [12]. Further assessment of the effects of targeting TREM2 with agonists and antagonists is important in the context of different stages of AD pathology.

Overall, the authors’ discoveries provide important new considerations to our approaches for therapeutically targeting TREM2, open new avenues to consider in advancing AD therapeutics and raise several interesting questions for further study. What is it about the H157Y mutation and relationship between microglia, TREM2, sTREM2 and Aβ that results in decreased Aβ when assessed at a late stage of disease? Qiao et al. show that the hom *Trem2*^H157Y/H157Y^ mice displayed increased production of sTREM2. It is certainly possible that the increase in sTREM2 is leading to some of these effects. What are the roles of TREM2 and sTREM2 on neuronal activity and function. How will the H157Y mutation and *Trem2*^R47H NSS^ model affect tau pathology and neurodegeneration? While these and other critical questions remain, the findings from these two exciting papers solidify the growing idea that the effects TREM2 and microglia are context dependent and differ based on disease severity, brain region, age, sex, mutation type and the types of pathologies present. All these factors need to be better studied and considered when designing TREM2 targeting therapies with the goal of mitigating AD and other CNS disease associated pathologies.

## Data Availability

Not applicable.

## Abbreviations

AD: Alzheimer’s disease
TREM2: Triggering Receptor Expressed on Myeloid Cells 2
ITAM: Immunoreceptor tyrosine-based activation motif
BAC: Bacterial artificial chromosome
NSS: Normal Splice Site
CSS: Cryptic Splice Site
Aβ: Amyloid beta
Hom: Homozygous
Het: Heterozygous
WT: Wild Type
sTREM2: Soluble TREM2
